# Hepatitis B in Isolated Anti-HBc Carriers: Predictors of Detectable HBV DNA and Implications for Risk-Stratified Screening

**DOI:** 10.3390/v18080899

**Published:** 2026-08-14

**Authors:** Hasan Zeybek

**Affiliations:** Department of Medical Microbiology, Gulhane Training and Research Hospital, 06010 Ankara, Türkiye; hsnzeybek86@gmail.com; Tel.: +90-541-433-9686

**Keywords:** isolated anti-HBc, HBV DNA, number needed to screen, diagnostic stewardship, immunosuppression, HBV reactivation

## Abstract

**Background/Objectives:** Isolated anti-HBc (IAHBc)—anti-HBc reactivity without HBsAg or anti-HBs—may indicate occult HBV infection (OBI) and confers reactivation risk under immunosuppression, yet most evidence derives from single specialties. We assessed the frequency, predictors, and diagnostic yield of detectable HBV DNA across multiple clinical specialties in a large real-world cohort to inform risk-based testing. **Methods:** In this 8-year, single-center retrospective study from an intermediate-endemic setting, 36,506 patients were screened for HBsAg, anti-HBs, and anti-HBc IgG. Patients with the IAHBc pattern who underwent HBV DNA testing formed the analytic cohort. Predictors of HBV DNA positivity were identified by multivariable logistic regression, and the number needed to screen (NNS) was calculated per specialty. **Results:** IAHBc prevalence was 4.16% (1517/36,506); 1412 patients comprised the analytic cohort (median age 60 years; 55.2% male). HBV DNA was detectable in 117 patients (8.3% of the cohort; 0.32% of all screened). Positivity varied more than fifteen-fold across specialties, with NNS ranging from 4.3 in gastroenterology to 66 in other clinics. High-intensity immunosuppression was the strongest independent predictor (adjusted OR 14.5), alongside younger age and gastroenterology or infectious diseases referral; the model showed good discrimination (AUC 0.810), while male sex was not an independent predictor. Among positive patients, 47% had HBV DNA below 200 IU/mL, consistent with low-level occult infection. **Conclusions:** These findings support guideline-recommended HBV DNA testing in all anti-HBc-positive patients before immunosuppression and, where universal testing is not feasible, provide local denominators for prioritizing it as an exercise in diagnostic stewardship. The proposed algorithm is institution-level rather than generalizable.

## 1. Introduction

Hepatitis B virus (HBV) infection remains a major global health problem and a leading cause of chronic liver disease, cirrhosis, and hepatocellular carcinoma [1,2]. Serological screening for HBV relies primarily on three markers: hepatitis B surface antigen (HBsAg), antibody to surface antigen (anti-HBs), and antibody to core antigen (anti-HBc). Among the serological profiles encountered in clinical practice, the isolated anti-HBc pattern—defined as positive anti-HBc IgG in the absence of both HBsAg and anti-HBs—is the most common atypical profile and poses a recurrent interpretive challenge for clinicians and laboratory physicians alike [3,4,5].

Anti-HBc reactivity may manifest in three serologically distinct patterns: HBsAg positivity, indicating current HBV infection; anti-HBs positivity, signifying resolved infection with intact humoral immunity; and the isolated anti-HBc pattern (IAHBc), defined by anti-HBc reactivity in the absence of both HBsAg and anti-HBs [6,7,8]. The clinical interpretation of IAHBc is inherently complex, as this profile may reflect a spectrum of underlying conditions: late-resolved infection with natural waning of anti-HBs titers, the diagnostic window period of acute infection, false-positive serology, or—most consequentially—occult HBV infection (OBI) [9,10]. Once the acute window period has been excluded, IAHBc effectively narrows to one of two clinically divergent states: a serological scar of remote, resolved infection without ongoing viral replication, or persistent OBI. OBI is formally defined as the presence of HBV DNA in serum or liver tissue in HBsAg-negative individuals, with or without detectable anti-HBc and/or anti-HBs [9,10,11]. Within this definition a distinction is drawn between true and false OBI. True OBI reflects genuine suppression of viral replication and transcription and is characterized by very low serum HBV DNA levels, usually below 200 IU/mL. False OBI denotes HBsAg-negative infection in which replication is not suppressed and HBV DNA levels are comparable to those of overt chronic infection. In this situation HBsAg is not absent but undetected, because diagnostic-escape mutations in the S gene alter the “a” determinant and impair recognition by commercial HBsAg immunoassays [10,12]. The distinction is clinically consequential, since false OBI carries the replicative burden and transmission potential of overt infection while presenting with an apparently resolved or isolated anti-HBc serological profile. Because the viral loads that characterize OBI are typically very low, often falling below the detection threshold of routine assays, its identification depends critically on the analytical sensitivity of the HBV DNA testing platform employed [12]. Distinguishing true occult infection from a serological scar therefore carries direct implications for both diagnostic interpretation and subsequent clinical management.

The clinical relevance of IAHBc is disproportionate to its modest population prevalence, particularly in the immunocompromised host. Patients receiving B-cell-depleting therapies—notably anti-CD20 monoclonal antibodies such as rituximab, undergoing hematopoietic stem-cell transplantation, or receiving anti-tumor necrosis factor (anti-TNF) agents—face a tangible risk of HBV reactivation that may culminate in fulminant hepatitis, hepatic decompensation, or death [13,14,15]. The magnitude of this risk is drug-dependent: a large systematic review and meta-analysis of nearly 5800 HBsAg-negative, anti-HBc-positive patients stratified reactivation risk by drug class and identified B-cell-depleting agents as carrying the highest risk [16], with rituximab associated with reactivation rates of up to 50–64% in some series, irrespective of underlying disease [17]. Reflecting this evidence, the 2024–2025 update of the American Gastroenterological Association (AGA) clinical practice guideline stratifies HBV reactivation risk into high (>10%), moderate (1–10%), and low (<1%) categories on the basis of the pre-treatment serological profile and the immunosuppressive regimen administered, with explicit recommendations for antiviral prophylaxis in moderate to high-risk strata [14]. Risk is conditioned first by baseline serological status and only then by the regimen. For any given exposure, HBsAg-positive patients carry a substantially higher reactivation risk than HBsAg-negative, anti-HBc-positive patients; in the estimates underpinning the AGA guideline, for example, anti-TNF exposure was associated with a reactivation risk of approximately 33% in HBsAg-positive patients but below 1% in HBsAg-negative, anti-HBc-positive patients [14]. Within the HBsAg-negative, anti-HBc-positive population the pooled reactivation rate is approximately 6.5%, but a separate systematic review stratifying by anti-HBs status showed that this average conceals a strong gradient. Patients lacking anti-HBs, which is precisely the isolated anti-HBc phenotype examined here, showed reactivation rates roughly three-fold higher than anti-HBs-positive patients: 21.8% versus 7.1% in hematological disease, 19.8% versus 6.6% under rituximab-containing regimens, and 10.7% versus 2.5% in non-hematological disease [18]. Isolated anti-HBc therefore occupies an intermediate position, at lower risk than HBsAg positivity but consistently the highest-risk category among HBsAg-negative individuals, and at a level that falls within the high-risk stratum defined above when B-cell-depleting agents are used.

Consistent with this, the EASL and AASLD guidelines recommend serological screening for HBsAg, anti-HBc IgG, and anti-HBs before immunosuppressive therapy in regions where HBsAg prevalence exceeds 2%, with HBV DNA testing indicated when anti-HBc is positive [19,20]. Despite the growing weight of these recommendations, the true prevalence of IAHBc—and the fraction harboring genuine occult HBV replication—across high-risk patient populations in intermediate-endemic settings remains incompletely characterized, leaving clinicians and laboratories without robust local denominators to guide risk-stratified screening.

Turkey lies within an intermediate-endemic region, with a reported HBsAg positivity rate of approximately 4% and an anti-HBc positivity rate of around 30%, rendering the interpretation of isolated anti-HBc particularly relevant in this setting [21,22]. Despite the recognized importance of this serological pattern, most published studies have focused on single patient populations—predominantly hematology or rheumatology cohorts—and few have systematically compared the determinants of detectable HBV DNA across multiple clinical specialties within a single institution. Moreover, the diagnostic yield of universal HBV DNA testing in all isolated anti-HBc carriers, as opposed to a targeted, risk-stratified approach, remains insufficiently characterized. Reported HBV DNA positivity rates in isolated anti-HBc carriers vary widely depending on assay sensitivity and population characteristics, with recent regional data reporting rates of approximately 8% [23].

Against this background, we conducted an 8-year retrospective analysis in a tertiary care setting to determine the frequency of detectable HBV DNA among patients with isolated anti-HBc and to identify the demographic, clinical, and specialty-related factors associated with HBV DNA positivity. By examining these determinants across multiple medical specialties and stratifying by immunosuppression intensity, we aimed to provide a laboratory-based, risk-stratified framework to inform HBV DNA testing decisions in this population.

## 2. Materials and Methods

### 2.1. Study Design and Setting

This single-center retrospective observational study was conducted at Gulhane Training and Research Hospital, a tertiary university-affiliated hospital in Ankara, Turkey. Electronic medical records of patients tested for HBV serological markers were reviewed. The laboratory information system was searched to identify all adult patients (≥18 years) tested for HBsAg, anti-HBs, and anti-HBc IgG over an 8-year period 1 January 2017–31 December 2024. The study was reported in accordance with the STROBE guidelines for observational studies [20].

### 2.2. Inclusion and Exclusion Criteria

Among 36,506 patients screened, those meeting the definition of isolated anti-HBc—negative HBsAg, negative anti-HBs, and positive anti-HBc IgG—and who had undergone HBV DNA PCR testing were eligible for inclusion.

Adult patients (≥18 years) followed in the Departments of Internal Medicine clinics including Gastroenterology, Rheumatology, Hematology, Medical Oncology, or Infectious Diseases and Clinical Microbiology and other departments during the study period, with simultaneous testing for HBsAg, anti-HBs, and anti-HBc, were eligible for study.

Exclusion criteria were: (i) duplicate records (only the first encounter retained); (ii) missing identifier or critical demographic data; (iii) age < 18 years; (iv) positive anti-HBs or negative anti-HBc IgG; (v) ongoing antiviral or nucleos(t)ide analogue therapy at the time of testing; (vi) HIV or HCV co-infection.

Patients with HIV or HCV co-infection were excluded, as these conditions are independently associated with occult HBV infection and altered HBV DNA kinetics, which could confound the assessment of specialty and immunosuppression-related predictors. The final analysis included 1412 patients.

### 2.3. Definitions and Laboratory Methods

Serological patterns were defined as follows. IAHBc: HBsAg-negative (S/CO < 1.0); anti-HBs negative (<10 mIU/mL); anti-HBc (IgG) positive. Resolved HBV infection: HBsAg negative; anti-HBs positive and anti-HBc positive. Occult HBV infection was defined as detectable HBV DNA in the plasma of an HBsAg negative (IAHBc) patient, consistent with the Taormina criteria; serum HBV DNA in OBI is typically low (often <200 IU/mL) [10]. Serological HBV assays (HBsAg, anti-HBs quantitative titer, anti-HBc total) were analyzed from serum samples by chemiluminescent microparticle immunoassay (CMIA) using the Cobas e801 analyzer (Roche Diagnostics, Mannheim, Germany) and the Architect i2000SR system (Abbott Diagnostics, Abbott Park, IL, USA). Anti-HBs levels < 10 IU/mL, HBsAg index values < 1.00 signal to cutoff (S/CO), and anti-HBc values < 1.00 S/CO were considered negative. For anti-HBc testing, results obtained with the Architect i2000SR platform were interpreted as negative when the S/CO value was <1.00. In contrast, due to the competitive immunoassay design of the Cobas e801 system, anti-HBc values < 1.00 S/CO were accepted as positive in accordance with the manufacturer’s instructions. In the present study both conditions were addressed as far as routine diagnostic practice permits. HBsAg was determined by qualitative chemiluminescent immunoassay with a manufacturer-declared analytical sensitivity of approximately 0.05 IU/mL against the WHO International Standard, and HBV DNA by a real-time PCR assay with a lower limit of quantification of 10 IU/mL. Quantitative HBsAg measurement, confirmatory testing on a second platform with a different antibody pair, and S-gene sequencing were not available, however, so misclassification arising from diagnostic-escape variants cannot be formally excluded, and reported OBI frequencies from different centers remain only partially comparable for this reason.

HBV DNA was quantified by real-time quantitative PCR. Viral amplification was performed on plasma specimens using the Montania 4896 Real-Time PCR platform (Anatolia Geneworks, Istanbul, Türkiye) with a linear quantification range of 10 to 1 × 10^9^ IU/mL, so that 10 IU/mL represents the lower limit of quantification. Specimens that generated a reproducible amplification signal at a concentration below this threshold were reported as HBV DNA detected, below the limit of quantification. Such results are qualitatively positive, it means “<LOQ” (detectable but not quantifiable viral load between >LOD and <LOQ). They were counted as HBV DNA-positive for the primary outcome and treated as left-censored at 10 IU/mL in all quantitative analyses. All serological and molecular parameters were interpreted according to the manufacturers’ recommended cut-off values.

### 2.4. Data Collection and Variable Definitions

Patient-level data were extracted from the hospital information system and laboratory information management system covering the study period (January 2017–December 2024). The following variables were systematically retrieved for each subject. The requesting clinical specialty was categorized as Rheumatology, Hematology, Oncology, Gastroenterology, Infectious Diseases, Nephrology, or other internal medicine departments. Eligibility for the analytic cohort was defined by the availability of an HBV DNA result, whether detectable or undetectable, and not by the result itself. HBV DNA detectability was the outcome of the study, not a selection criterion. All patients with the isolated anti-HBc pattern in whom HBV DNA testing had been performed during the study period were therefore included and were subsequently stratified into an HBV DNA-detectable group and an undetectable comparison group. Patients with detectable HBV DNA constituted the HBV DNA-detectable group, whereas patients with undetectable HBV DNA served as the comparison group. Independent variables included age, sex, requesting specialty, underlying disease category, and immunosuppressive therapy status.

Virological data, where available, comprised HBV DNA detection status and quantitative viral load (IU/mL, log_10_-transformed for analysis). Co-infection status was ascertained through anti-HCV and anti-HIV serology. Referring departments were grouped into the following categories used throughout the analysis: Gastroenterology, Infectious Diseases, Hematology, Oncology, Rheumatology, Nephrology, and Other (the latter encompassing Internal Medicine, Neurology, Intensive Care, Dermatology, and remaining specialty services). For the multivariable regression model only, Nephrology was combined with the Other category to ensure model stability; this is also indicated in the footnote to the corresponding table. Because individual pharmacological records were not available for the entire cohort owing to the retrospective design, immunosuppression intensity was classified dichotomously as high versus moderate/low, using a proxy based on treating specialty and primary diagnosis. The high-intensity group comprised patients receiving B-cell-depleting agents (e.g., rituximab) or intensive cytotoxic/antineoplastic chemotherapy or undergoing hematopoietic stem-cell transplantation; all remaining patients were classified as moderate/low.

Primary diagnoses were classified according to the International Classification of Diseases, 10th Revision (ICD-10), and subsequently aggregated into clinically meaningful disease groups (e.g., solid malignancy, hematological malignancy, rheumatological disease, inflammatory disease) for downstream analysis.

### 2.5. Statistical Analysis

Statistical analyses were performed using GraphPad Prism version 11.0.1 (GraphPad Software, San Diego, CA, USA) and IBM SPSS Statistics version 26.0 (IBM Corp., Armonk, NY, USA). Continuous variables were assessed for normality with the Shapiro–Wilk test and compared using the Student *t*-test or Mann–Whitney U test as appropriate; results are presented as mean ± SD or median (IQR). Categorical variables, expressed as *n* (%), were compared using the chi-square or Fisher’s exact test.

Variables with *p* < 0.20 in univariate analysis, together with clinically relevant covariates, were entered simultaneously into a multivariable logistic regression model (enter method). Results are reported as odds ratios (OR) with 95% confidence intervals (CIs). Proportions are presented with 95% Wilson confidence intervals. The number needed to screen (NNS) was calculated as the reciprocal of the HBV DNA positivity rate, with 95% confidence intervals derived as the reciprocals of the corresponding Wilson interval bounds. Model calibration was evaluated with the Hosmer–Lemeshow test, explanatory power with Nagelkerke R^2^, and discrimination with the area under the ROC curve (AUC). The 95% confidence interval for the AUC was estimated by bootstrap re-sampling with 2000 replications. Statistical significance was set at *p* < 0.05.

### 2.6. Ethics

The study was conducted in accordance with the Declaration of Helsinki, and the protocol was approved by the Ethics Committee of Health Sciences University Gulhane Scientific Research (decision no: 2025-454) on 30 September 2025. Given the retrospective design and use of fully anonymized data, the requirement for individual informed consent was waived by the Ethics Committee.

## 3. Results

### 3.1. Patient Characteristics and Cohort Assembly

**Figure 1:**
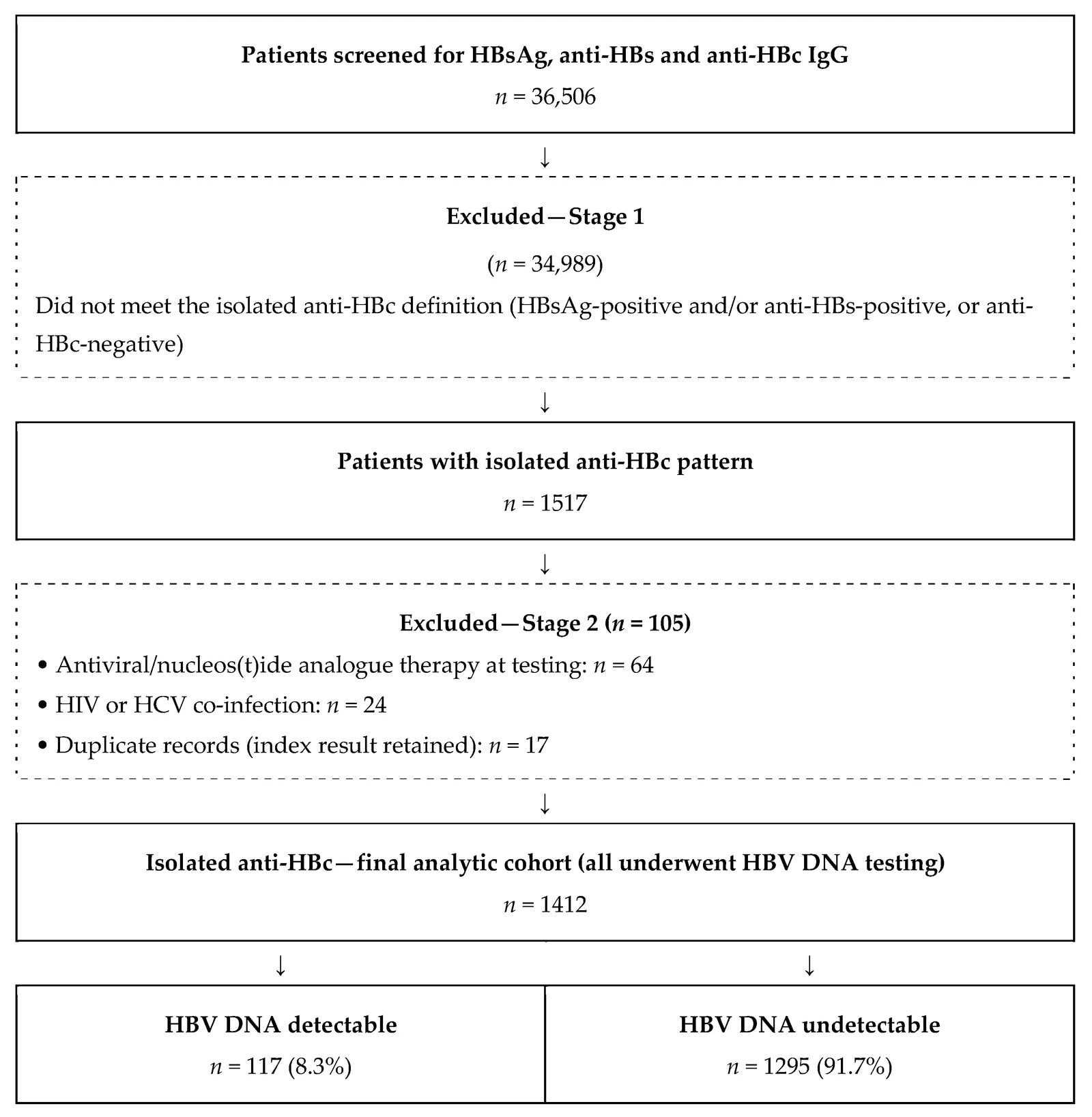
Flow diagram of patient selection. Of 36,506 patients screened with a complete HBV serological panel, 1517 showed the isolated anti-HBc pattern; after stage-2 exclusions, 1412 comprised the final analytic cohort, of whom 117 (8.3%) had detectable HBV DNA.

During the study period, 36,506 patients were screened with a complete HBV serological panel. Of these, 34,989 did not meet the definition of isolated anti-HBc (HBsAg-positive and/or anti-HBs-positive, or anti-HBc-negative) and were excluded, leaving 1517 patients with the isolated anti-HBc pattern. A further 105 patients were excluded: 64 were receiving antiviral or nucleos(t)ide analogue therapy at the time of testing, 24 had HIV or HCV co-infection, and 17 were duplicate records (for whom the index result was retained). The final analytic cohort comprised 1412 patients, all of whom underwent HBV DNA testing (Figure 1). The median age was 60 years (IQR 50–70) and 780 (55.2%) were male. Patients were distributed across requesting specialties as follows: Other clinics (*n* = 465, 32.9%), Rheumatology (*n* = 271, 19.2%), Infectious Diseases (*n* = 217, 15.4%), Oncology (*n* = 168, 11.9%), Gastroenterology (*n* = 158, 11.2%), Hematology (*n* = 104, 7.4%), and Nephrology (*n* = 29, 2.1%). Baseline characteristics of the cohort, stratified by HBV DNA status, are presented in Table 1.

### 3.2. Frequency of Detectable HBV DNA

HBV DNA was detectable in 117 of 1412 patients, corresponding to an overall positivity of 8.3% (95% CI 7.0–9.8%). The overall number needed to screen (NNS) to detect one HBV DNA-positive patient was 12.1 (95% CI 10.2–14.4).

Baseline characteristics stratified by HBV DNA status are presented in Table 1. DNA-positive patients were significantly younger than DNA-negative patients (median 55 (IQR 41–65) vs. 59 (IQR 49–66) years; Mann–Whitney U *p* = 0.019). Male sex was significantly more frequent in the DNA-positive group (70.1% vs. 53.9%; *p* < 0.001). The distribution of requesting specialties also differed significantly between groups (*p* < 0.001), with Infectious Diseases and Gastroenterology over-represented among DNA-positive patients (38.5% and 31.6%, respectively).

### 3.3. HBV DNA Positivity and Diagnostic Yield by Specialty

HBV DNA positivity rates differed significantly across requesting specialties (chi-square *p* < 0.001; Table 2). The highest rates were observed in Gastroenterology (37/158, 23.4%; 95% CI 17.5–30.6%) and Infectious Diseases (45/217, 20.7%; 95% CI 15.9–26.6%), whereas Rheumatology (10/271, 3.7%; 95% CI 2.0–6.7%), Nephrology (1/29, 3.4%; 95% CI 0.6–17.2%), and Other clinics (7/465, 1.5%; 95% CI 0.7–3.1%) showed the lowest rates.

**Figure 2:**
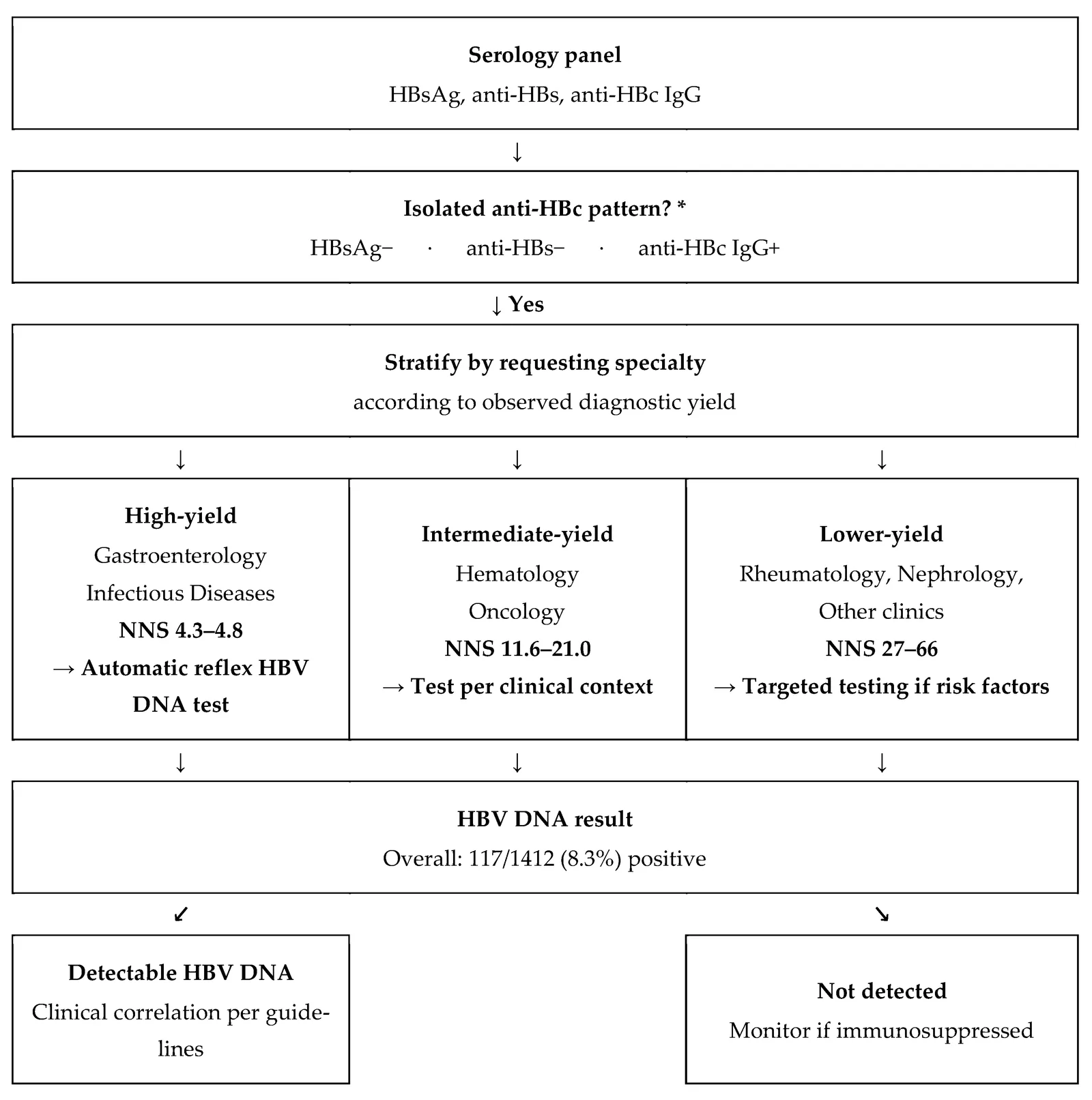
Proposed reflex-testing algorithm for the isolated anti-HBc pattern, stratified by requesting specialty according to observed diagnostic yield (Table 2). Yield strata are those defined in Section 3.3 (high-yield, NNS below 5; intermediate-yield, NNS 5 to 25; lower-yield, NNS above 25). NNS was lowest, that is, yield highest, in Gastroenterology (4.3) and Infectious Diseases (4.8) and highest in lower-yield clinics (up to 66). Because HBV DNA was ordered at physician discretion, specialty-level yield may partly reflect indication bias and should be interpreted accordingly. The algorithm reflects the case mix and referral patterns of a single tertiary center and is intended as a locally calibrated implementation tool. It does not replace the guideline recommendation to test HBV DNA in all anti-HBc-positive patients before immunosuppression, and its yield strata would require local re-derivation in other settings. * If the pattern is not isolated anti-HBc (other serological profile): standard workup—no reflex HBV DNA testing indicated.

The corresponding NNS values ranged from 4.27 (95% CI 3.3–5.7) in Gastroenterology and 4.82 (95% CI 3.8–6.3) in Infectious Diseases to 27.10 (95% CI 15.0–49.6) in Rheumatology, 29.00 (95% CI 5.8–163.6) in Nephrology, and 66.43 (95% CI 32.5–136.8) in Other clinics (Table 2). Hematology and Oncology yielded intermediate NNS estimates of 11.56 (95% CI 6.4–21.6) and 21.00 (95% CI 11.0–41.1), respectively. For descriptive purposes, and solely on the basis of the yields observed in this cohort, requesting specialties were grouped post hoc into three strata according to the number needed to screen: high-yield (NNS below 5: Gastroenterology and Infectious Diseases), intermediate-yield (NNS 5 to 25: Hematology and Oncology) and lower-yield (NNS above 25: Rheumatology, Nephrology and Other clinics). These labels are descriptive summaries of the observed gradient in the present data rather than externally validated thresholds and are used in this sense throughout the Discussion and in Figure 2.

### 3.4. Predictors of Detectable HBV DNA

Univariable analysis. In univariable logistic regression, older age was inversely associated with HBV DNA positivity (OR 0.968 per year, 95% CI 0.956–0.980; *p* < 0.001). Male sex was also associated (OR 1.895, 95% CI 1.257–2.857; *p* = 0.002). High-intensity immunosuppression was the strongest univariable predictor (OR 8.922, 95% CI 4.463–17.833; *p* < 0.001). Compared with the Other clinics reference category, significant associations were found for Gastroenterology (OR 11.619, 95% CI 5.466–24.699; *p* < 0.001), Infectious Diseases (OR 9.047, 95% CI 4.346–18.831; *p* < 0.001), and Hematology (OR 4.744, 95% CI 1.832–12.286; *p* = 0.001). Oncology reached statistical significance at the univariable level (OR 2.654, 95% CI 1.005–7.009; *p* = 0.049), while Rheumatology did not (OR 1.883, 95% CI 0.755–4.696; *p* = 0.175). Full results are presented in Table 3.

Multivariable analysis. In multivariable logistic regression analysis (Table 3), high-intensity immunosuppression remained the strongest independent predictor (adjusted OR (aOR) 14.533, 95% CI 6.222–33.944; *p* < 0.001). Older age retained its inverse association (aOR 0.969 per year, 95% CI 0.956–0.983; *p* < 0.001). Male sex was not independently significant after adjustment (aOR 1.311, 95% CI 0.840–2.045; *p* = 0.233). Among specialties, Gastroenterology (aOR 10.705, 95% CI 4.960–23.103; *p* < 0.001) and Infectious Diseases (aOR 7.541, 95% CI 3.581–15.879; *p* < 0.001) remained independently associated with HBV DNA positivity. Hematology reached borderline significance (aOR 2.827, 95% CI 1.001–7.979; *p* = 0.050), whereas Oncology (aOR 1.401, 95% CI 0.473–4.151; *p* = 0.542) and Rheumatology (aOR 1.592, 95% CI 0.621–4.083; *p* = 0.333) did not.

The multivariable model showed good discriminatory ability, with an area under the receiver operating characteristic curve of 0.810 (95% CI 0.770–0.845; Figure S1). The model was statistically significant overall, as indicated by the likelihood-ratio test (*p* < 0.001), and explained a moderate proportion of the variance in HBV DNA positivity, as reflected by a Nagelkerke R^2^ of 0.218. Model calibration was adequate, as the Hosmer–Lemeshow test was not statistically significant (χ^2^ = 9.60, *p* = 0.294). Overall, high-intensity immunosuppression and management within the Gastroenterology or Infectious Diseases services were independently associated with higher odds of detectable HBV DNA, whereas older age was associated with lower odds, among patients with isolated anti-HBc positivity.

### 3.5. Quantitative HBV DNA Analysis

Among the 117 DNA-positive patients, quantifiable viral loads ranged from 10 IU/mL to 6.7 × 10^8^ IU/mL (median 242 IU/mL, IQR 34–4200 IU/mL). Eight patients (6.8%) had HBV DNA detected below the lower limit of quantification (Table 4). These results are qualitatively positive, in that HBV DNA was reproducibly amplified, but the concentration was too low to be quantified reliably; they were therefore retained as HBV DNA-positive and treated as left-censored at 10 IU/mL, so that the reported median and interquartile range are conservative estimates. Overall, 55 patients (47.0%)—comprising those below the limit of quantification and those with low-level viremia—had HBV DNA below 200 IU/mL, consistent with low-level occult infection. In contrast, 37 patients (31.6%) had viral loads ≥ 2000 IU/mL, a level at which clinically significant replication and a higher risk of reactivation are more likely. Notably, among DNA-positive patients, viral load magnitude did not differ by immunosuppression status (median 242 vs. 247 IU/mL in patients on high- versus moderate/low-intensity immunosuppression; *p* = 0.794), and the proportion with ≥2000 IU/mL was comparable between these groups (33% vs. 31%), indicating that immunosuppression was associated with the presence rather than the magnitude of detectable viremia.

## 4. Discussion

This 8-year retrospective analysis represents one of the largest single-center characterizations of hepatitis B serology and isolated anti-HBc reactivity from an intermediate-endemic setting, encompassing 36,506 unique patients screened simultaneously for the three core HBV markers. Five principal findings emerge from our data. First, the prevalence of isolated anti-HBc was 4.16% (1517/36,506), concordant with Türkiye’s intermediate-endemicity classification. Second, detectable HBV DNA was found in 8.3% of the analyzed IAHBc cohort (117/1412; 0.32% of all those screened), of whom approximately half had low-level viremia (<200 IU/mL) consistent with occult infection. Third, the IAHBc cohort was older (median 60 years) and modestly male-predominant (55.2%). Fourth, the clinical profile of IAHBc patients was dominated by referrals from Internal Medicine and high-yield specialties, with malignancies representing a prominent high-risk diagnostic indication. Fifth, multivariable logistic regression identified high-intensity immunosuppression as the strongest independent predictor of detectable HBV DNA (aOR 14.5), together with younger age and management within the Gastroenterology or Infectious Diseases services, whereas male sex was not independently associated with OBI after adjustment—a pattern with direct implications for risk-stratified HBV DNA testing.

In this tertiary care cohort of 1412 patients with isolated anti-HBc seropositivity, HBV DNA was detectable in 117 patients (8.3%). This rate falls within the range reported previously [11], the variability of which reflects differences in study populations, anti-HBc assay characteristics, and the analytical sensitivity of HBV DNA detection methods; notably, it closely matches the approximately 8% positivity reported in a comparable cohort from the same intermediate-endemic setting [23]. The observation that approximately one in twelve isolated anti-HBc carriers harbored detectable viremia indicates that this serological pattern is not uniformly indicative of resolved infection and may, in a definable subset, represent occult HBV infection with potential clinical relevance under immunosuppression.

Stratification by requesting specialty revealed substantial and statistically significant heterogeneity in HBV DNA detectability. Crude positivity was highest in Gastroenterology and Infectious Diseases, intermediate in Hematology and Oncology, and lowest in Rheumatology, Nephrology and the Other clinics group (Table 2). After adjustment for age, sex and immunosuppression intensity, Gastroenterology and Infectious Diseases retained strong independent associations with HBV DNA positivity, Hematology was of borderline significance, and neither Oncology nor Rheumatology was independently associated (Table 3). Specialty therefore carried information beyond immunosuppressive burden alone. The high adjusted ORs for Gastroenterology and Infectious Diseases likely reflect, at least in part, the preferential referral of patients with underlying liver disease or suspected HBV-related conditions to these specialties—a form of confounding by indication that should be interpreted cautiously and is addressed in the limitations. Nonetheless, the finding that specialty remained a significant predictor after covariate adjustment underscores its practical value as a stratifier in laboratory-based screening decisions. To our knowledge, this is among the few studies to systematically compare HBV DNA detectability across multiple clinical specialties within a single institutional cohort, providing a more granular, real-world risk profile than the single-specialty analyses that dominate the existing literature.

In multivariable analysis, high-intensity immunosuppression was by far the strongest independent predictor of detectable HBV DNA (aOR 14.5, 95% CI 6.2–33.9; *p* < 0.001, Table 3). This finding is consistent with the well-established pathophysiology of HBV reactivation: even after apparent serological resolution, transcriptionally active covalently closed circular DNA (cccDNA) persists within hepatocytes, and the loss of T-cell- and B-cell-mediated immune surveillance under immunosuppression permits renewed viral replication. The magnitude of this association—an approximately 14-fold increase in odds—despite the small proportion of patients receiving high-intensity regimens (2.5% of the cohort) underscores why pre-treatment identification of isolated anti-HBc carriers is emphasized in current guidelines: both EASL and AASLD recommend HBV DNA assessment and risk-based antiviral prophylaxis in anti-HBc-positive patients before B-cell-depleting agents, intensive chemotherapy, or solid-organ and hematopoietic transplantation [19,24,25]. From a virological standpoint, the predominance of low-level viremia in our cohort—47% of positive patients had HBV DNA below 200 IU/mL—reflects the characteristically minimal replication of occult infection and reinforces the need for sensitive nucleic acid testing to avoid false negative results in this at-risk population, although the substantial minority of patients with considerably higher viral loads requires a different interpretation, considered below. Of note, while high-intensity immunosuppression was strongly associated with the presence of detectable HBV DNA, it was not associated with the magnitude of viremia among positive patients, suggesting that impaired immune control primarily determines whether occult replication becomes detectable rather than how high the viral load rises in this cross-sectional setting.

The diagnostic yield of HBV DNA testing varied markedly across clinical contexts, with the number needed to screen (NNS) to detect one viremic patient ranging from approximately 4–5 in Gastroenterology and Infectious Diseases to 27 in Rheumatology and 66 in Other clinics—an almost 15-fold difference. From an infectious disease perspective, these gradients help define where targeted HBV DNA testing is most likely to identify clinically relevant occult infection and, in turn, which isolated anti-HBc carriers warrant closer virological monitoring for reactivation once immunosuppression is initiated. Where universal HBV DNA testing cannot be delivered, a risk-stratified approach, prioritizing patients on high-intensity immunosuppression and those managed within the high-yield specialties defined above, identifies the individuals at greatest risk soonest. Importantly, the high yield observed in Gastroenterology and Infectious Diseases likely reflects, at least in part, confounding by indication: HBV DNA was ordered at clinician discretion, and these specialties preferentially evaluate patients with established or suspected liver disease. This pattern should therefore be interpreted as real-world testing behavior rather than an intrinsically higher reactivation risk, and it argues for embedding HBV DNA assessment into standardized, indication-driven pathways rather than relying on ad hoc requests. Such a framework—triggering HBV DNA testing on the basis of the isolated anti-HBc pattern combined with immunosuppression status—could improve the timely detection of occult infection and support guideline-concordant reactivation prophylaxis. This approach is also conducive to a laboratory-driven reflex-testing algorithm, in which the isolated anti-HBc serological pattern combined with clinical metadata automatically triggers HBV DNA testing in high-yield contexts, shifting practice from an inconsistent, clinician-dependent request pattern toward a standardized, evidence-based laboratory pathway (Figure 2). The feasibility of this approach depends on the transmission of adequate clinical information with the test request, underscoring the pre-analytical importance of complete request metadata. Additionally, because isolated anti-HBc reactivity carries a non-negligible false-positivity rate, confirmatory testing of reactive results may be warranted before triggering reflex HBV DNA analysis in low-risk subgroups where the pre-test probability of genuine occult infection is low [26].

Scope and applicability of the proposed algorithm. Current guidance is unambiguous. EASL, AASLD and the 2025 AGA guideline all recommend screening for HBsAg, anti-HBc and anti-HBs before immunosuppressive therapy, with HBV DNA testing in every anti-HBc-positive patient irrespective of the referring service [14,19,24]. Our findings do not modify that recommendation, and the algorithm shown in Figure 2 is not offered as an alternative to it. Where guideline-concordant testing is fully implemented, the requesting specialty has no independent decision weight, since every immunosuppressed isolated anti-HBc carrier is tested in any case. The specialty-stratified yield data reported here therefore serve three narrower purposes. First, they describe actual testing behavior in a real-world tertiary center and quantify how far it departs from a uniform, guideline-driven pattern. Second, they distinguish contexts that superficially resemble one another: the very low yield in the Other clinics group reflects testing requested outside any recognized indication, whereas the comparably low yield in Rheumatology reflects appropriate protocol-driven screening of asymptomatic candidates for biologic therapy, and the two carry opposite implications for practice. Third, and only in settings where universal HBV DNA testing of all anti-HBc-positive patients is not financially or logistically achievable, the observed gradient offers an empirical basis for deciding the order in which implementation should proceed. We present the algorithm accordingly as a locally calibrated, resource-dependent implementation tool for a single institution rather than as a generalizable screening recommendation. Its parameters, namely the composition of the Other clinics group, the referral patterns of individual services and the resulting NNS values, are properties of this hospital’s case mix and organization and would require local re-derivation before use elsewhere.

Our findings are broadly consistent with prior reports on isolated anti-HBc and occult HBV infection. Davarcı et al. [23] documented detectable HBV DNA in approximately 8% of isolated anti-HBc carriers in a comparable cohort from the same intermediate-endemic setting, while Cai et al. [27] reported approximately 5% among HBsAg-negative, anti-HBc-positive individuals. However, most prior work has been confined to single-specialty cohorts—predominantly hematology or rheumatology populations—and has not compared the diagnostic yield of HBV DNA testing across multiple clinical specialties. The present analysis addresses this gap through a multispecialty, real-world cohort in which the yield of testing was quantified by requesting specialty and immunosuppression status, providing practical denominators for risk-stratified screening. Importantly, reported HBV DNA positivity rates in this population vary widely with assay sensitivity, and conventional assays may underestimate occult infection given its characteristically low viral loads; the use of a real-time PCR assay with a lower limit of quantification of 10 IU/mL minimizes this risk and strengthens the comparability of our estimates.

The predominance of low-level viremia should not be taken to mean that every DNA-positive patient in this cohort had true occult infection. The 37 patients (31.6% of DNA-positive cases) with HBV DNA of 2000 IU/mL or above are not typical of true OBI. In this subgroup the possibility of false OBI, that is, replicative HBsAg-negative infection caused by S-gene diagnostic-escape variants that are not recognized by the HBsAg immunoassays in routine use, cannot be excluded, because neither confirmatory HBsAg testing on an alternative platform nor S-gene sequencing was performed. Launay et al. described precisely this pattern, reporting high serum HBV DNA attributable to HBsAg mutants among patients with an anti-HBc alone profile [12]. The distinction is not academic: patients in this category require management as overt chronic hepatitis B rather than as occult infection, and their inclusion within an OBI denominator would misrepresent both the natural history and the transmission risk of the group.

The operational diagnosis of OBI is method-dependent in two respects, since it requires simultaneously that HBsAg be undetectable and that HBV DNA be detectable, and each condition is conditioned on assay performance. An HBsAg assay of limited analytical sensitivity, or one whose antibody pair recognizes S-escape variants poorly, will classify replicative infection as HBsAg-negative and thereby inflate the apparent frequency of occult infection. Conversely, an HBV DNA assay of limited analytical sensitivity will fail to detect the very low viral loads that characterize true OBI and will underestimate it [10,11]. In the present study both conditions were addressed as far as routine diagnostic practice permits. HBsAg was determined on high-sensitivity chemiluminescent platforms with a declared analytical sensitivity of approximately 0.05 IU/mL and HBV DNA by a real-time PCR assay with a lower limit of quantification of 10 IU/mL. Neither confirmatory HBsAg testing on a second platform nor S-gene sequencing was available, however, so misclassification arising from diagnostic-escape variants cannot be formally excluded, and reported OBI frequencies from different centers remain only partially comparable for this reason.

### Limitations

Several limitations warrant consideration. First, the retrospective, single-center design limits generalizability, and HBV DNA testing was performed at the discretion of treating physicians rather than under a standardized protocol. This introduces selection bias and, in particular, confounding by indication: the higher yield observed in Gastroenterology and Infectious Diseases likely reflects preferential testing of patients with suspected liver disease rather than an intrinsically higher risk, and the specialty-specific estimates should therefore be interpreted as reflecting real-world testing behavior. Second, immunosuppression was classified dichotomously (high versus moderate/low intensity), which precluded a graded, dose-dependent analysis and may have introduced residual misclassification. Third, the absence of longitudinal follow-up precluded assessment of subsequent reactivation events or clinical outcomes among DNA-positive patients. Fourth, quantitative anti-HBc titers were not systematically available, preventing evaluation of their potential value as a risk stratifier—an avenue worth prospective investigation [28]. Fifth, confirmatory anti-HBc testing was not routinely performed, so a proportion of isolated anti-HBc results may represent assay false positivity. Sixth, estimates for specialties with few patients (e.g., Nephrology) were imprecise, with wide confidence intervals, and should be interpreted with caution. Furthermore, although detectable HBV DNA in isolated anti-HBc carriers has implications for transfusion and transplantation safety, our study was not designed to assess transmission risk in these settings [29]. In addition, false OBI could not be excluded. HBsAg was determined by qualitative immunoassay only; quantitative HBsAg measurement, confirmatory testing on a second platform with a different antibody pair, and S-gene sequencing were not available. Patients whose HBsAg went undetected because of a diagnostic-escape mutation rather than genuinely suppressed replication would therefore have been classified as occult infection in this analysis. Finally, although HIV and HCV co-infected patients were excluded, residual confounding from other conditions affecting immune function cannot be entirely excluded. The large, multispecialty cohort and uniform laboratory methodology nonetheless support the robustness of the principal findings.

Beyond these methodological considerations, the external validity of the proposed algorithm is limited by design. Its yield strata derive from the referral structure and case mix of a single tertiary center, and the composition of the Other clinics group in particular is institution-specific. The algorithm is intended to support local implementation where resources constrain universal testing and should not be transferred to other settings without local re-derivation.

## 5. Conclusions

In conclusion, this 8-year analysis demonstrates that detectable HBV DNA is present in a clinically significant proportion—8.3%—of patients with isolated anti-HBc, confirming that this serological pattern does not uniformly represent resolved infection but may reflect latent HBV persistence with ongoing low-level replication. The likelihood of detectable viremia was independently and most strongly associated with high-intensity immunosuppression and varied markedly with the requesting clinical specialty. The high yield in Gastroenterology and Infectious Diseases settings most likely reflects an elevated pre-test probability among patients evaluated for suspected liver disease, whereas the low yield in Rheumatology is consistent with protocol-driven screening of largely asymptomatic carriers before immunosuppressive therapy. NNS estimates, differing more than fifteen-fold across specialties, indicate that the efficiency of testing varies markedly with clinical context. This does not displace the guideline recommendation to test HBV DNA in every anti-HBc-positive patient before immunosuppression; rather, it quantifies where that recommendation yields most and, in resource-constrained settings, the order in which it can realistically be implemented.

On this basis, and for institutions in which universal HBV DNA testing cannot yet be delivered, we propose a locally calibrated risk-stratified approach to HBV DNA testing in isolated anti-HBc carriers: prioritizing immediate HBV DNA quantification for patients about to receive high-intensity immunosuppression—such as B-cell-depleting agents or intensive chemotherapy—and for those evaluated in high-yield specialties such as gastroenterology, while maintaining the vigilant, protocol-based screening already standard before biologic therapy in rheumatology and other lower-yield settings. Embedding such specialty and immunosuppression-informed criteria into reflex-testing pathways could strengthen the early detection of clinically relevant occult infection, support guideline-concordant reactivation prophylaxis, and optimize molecular testing resources. These screening implications warrant confirmation in prospective, multicenter studies incorporating longitudinal follow-up of reactivation outcomes, which would also establish whether the specialty-level yield gradient observed here holds beyond a single institution.

## Figures and Tables

**Table 1:** Baseline characteristics of patients with isolated anti-HBc positivity according to HBV DNA status.

Characteristic	HBV DNA Positive (*n* = 117)	HBV DNA Negative (*n* = 1295)	*p*-Value
Age, median (IQR)	55.0 (41.0–65.0)	59.0 (49.0–66.0)	0.019
Sex			<0.001
Female	35 (29.9%)	597 (46.1%)	
Male	82 (70.1%)	698 (53.9%)	
Age ≥ 60 years			0.003
<60 years	71 (60.7%)	594 (45.9%)	
≥60 years	46 (39.3%)	701 (54.1%)	
**Branch/service group**			<0.001
Others	7 (6.0%)	458 (35.4%)	
Infectious Diseases	45 (38.5%)	172 (13.3%)	
Gastroenterology	37 (31.6%)	121 (9.3%)	
Hematology	9 (7.7%)	95 (7.3%)	
Nephrology	1 (0.9%)	28 (2.2%)	
Oncology	8 (6.8%)	160 (12.4%)	
Rheumatology	10 (8.5%)	261 (20.1%)	

Note: Data are presented as median (IQR) or *n* (%). Categorical variables were compared using chi-square or Fisher’s exact test, as appropriate. Age comparison was performed using the Mann–Whitney U test because age was non-normally distributed. IAHBc, isolated anti-HBc.

**Table 2:** Branch-specific HBV DNA positivity rate and number needed to screen among patients with isolated anti-HBc positivity.

Clinical Branch/Department	Total IAHBc Patients, *n*	HBV DNA Positive, *n*	Positivity Rate	95% CI	NNS	NNS 95% CI
Gastroenterology	158	37	23.4%	17.5–30.6%	4.27	3.3–5.7
Infectious Diseases	217	45	20.7%	15.9–26.6%	4.82	3.8–6.3
Hematology	104	9	8.7%	4.6–15.6%	11.56	6.4–21.6
Oncology	168	8	4.8%	2.4–9.1%	21.00	11.0–41.1
Rheumatology	271	10	3.7%	2.0–6.7%	27.10	15.0–49.6
Nephrology	29	1	3.4%	0.6–17.2%	29.00	5.8–163.6
Other clinics	465	7	1.5%	0.7–3.1%	66.43	32.5–136.8

Note: NNS was calculated as the reciprocal of the HBV DNA positivity rate. CI, confidence interval; IAHBc, isolated anti-HBc; NNS, number needed to screen. Estimates for branches with small denominators should be interpreted with caution.

**Table 3:** Univariable and multivariable logistic regression analyses of factors associated with detectable HBV DNA among patients with isolated anti-HBc positivity.

Variable	Univariable OR (95% CI)	*p*-Value	Multivariable aOR (95% CI)	*p*-Value
Age, per year	0.968 (0.956–0.980)	<0.001	0.969 (0.956–0.983)	<0.001
Male vs. female	1.895 (1.257–2.857)	0.002	1.311 (0.840–2.045)	0.233
High vs. Moderate/Low immunosuppression	8.922 (4.463–17.833)	<0.001	14.533 (6.222–33.944)	<0.001
Gastroenterology vs. Other clinics	11.619 (5.466–24.699)	<0.001	10.705 (4.960–23.103)	<0.001
Infectious Diseases vs. Other clinics	9.047 (4.346–18.831)	<0.001	7.541 (3.581–15.879)	<0.001
Hematology vs. Other clinics	4.744 (1.832–12.286)	0.001	2.827 (1.001–7.979)	0.050
Oncology vs. Other clinics	2.654 (1.005–7.009)	0.049	1.401 (0.473–4.151)	0.542
Rheumatology vs. Other clinics	1.883 (0.755–4.696)	0.175	1.592 (0.621–4.083)	0.333

Outcome variable: HBV DNA positivity. Reference branch/service group: Other clinics. OR, odds ratio; aOR, adjusted odds ratio; CI, confidence interval; AUC, area under the receiver operating characteristic curve. Model variables included age, sex, high-intensity immunosuppression, and clinical branch/service group. Nephrology was combined with other clinics for model stability. AUC = 0.810; Nagelkerke R^2^ = 0.218; Hosmer–Lemeshow χ^2^ = 9.60, *p* = 0.294; likelihood-ratio test *p* < 0.001.

**Table 4:** Distribution of quantitative HBV DNA levels among the 117 HBV DNA-positive patients with isolated anti-HBc.

Viral Load Category (IU/mL)	*n* (%)
<10 (below limit of quantification)	8 (6.8)
10–200	47 (40.2)
200–2000	25 (21.4)
≥2000	37 (31.6)
Median (IQR), IU/mL	242 (34–4200)

Data are presented as *n* (% of the 117 HBV DNA-positive patients) unless otherwise indicated. Values below 10 IU/mL were detected, below limit of quantification (LOQ) of the assay, and were treated as censored. Overall, 55 patients (47.0%) had HBV DNA < 200 IU/mL. IQR, interquartile range.

## Data Availability

The de-identified data presented in this study are available on reasonable request from the corresponding author. The data are not publicly available due to institutional and patient privacy considerations.

## Supplementary Materials

The following supporting information can be downloaded at: https://www.mdpi.com/article/10.3390/v18080899/s1, Figure S1: Receiver operating characteristic (ROC) curve of the multivariable logistic regression model for detectable HBV DNA in patients with isolated anti-HBc.
