# Supplementary material for: Hepatitis B in Isolated Anti-HBc Carriers: Predictors of Detectable HBV DNA and Implications for Risk-Stratified Screening
Source: Viruses. 2026 Aug 14;18(8):899. doi: 10.3390/v18080899 (PMC13517678; doi:10.3390/v18080899)
Supplement: Supplementary file 1 [file viruses-18-00899-s001.zip › viruses-4427755-supplementary.pdf]

Supplementary Materials

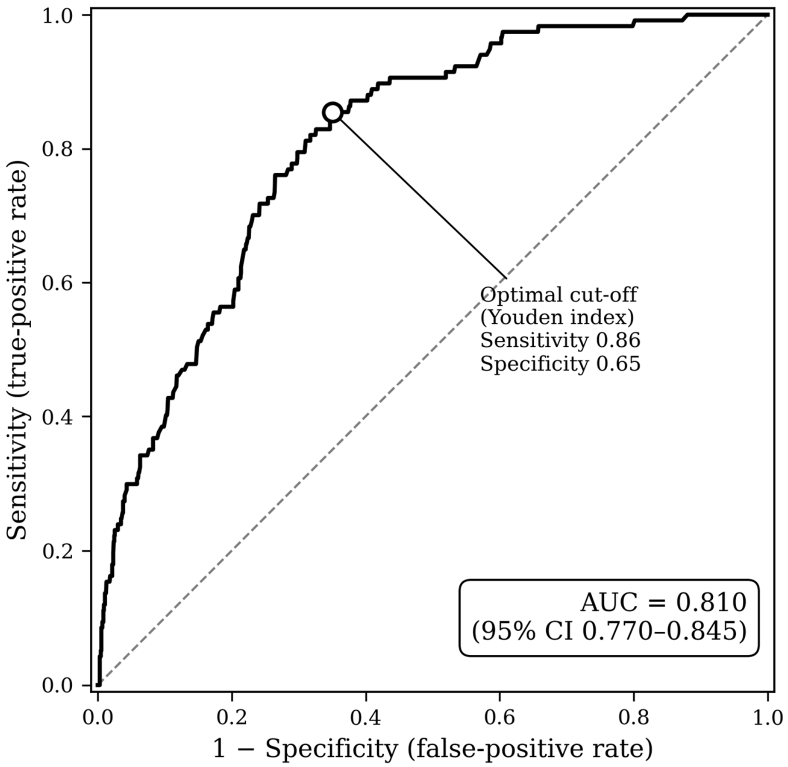

Figure S1. Receiver operating characteristic (ROC) curve of the multivariable logistic regression model for detectable HBV DNA in patients with isolated anti-HBc. The model (age, sex, high-intensity immunosuppression, and clinical branch) showed good discrimination (AUC = 0.810, 95% CI 0.770–0.845). The optimal cut-off by the Youden index corresponded to a sensitivity of 0.86 and a specificity of 0.65. The dashed diagonal represents the line of no discrimination.
